# Automated epileptic seizures detection using multi-features and multilayer perceptron neural network

**DOI:** 10.1186/s40708-018-0088-8

**Published:** 2018-09-03

**Authors:** N. Sriraam, S. Raghu, Kadeeja Tamanna, Leena Narayan, Mehraj Khanum, A. S. Hegde, Anjani Bhushan Kumar

**Affiliations:** 1 Centre for Medical Electronics and Computing, Ramaiah Institute of Technology (Affiliated to VTU Belgaum), Bengaluru, India; 2 Institute of Neuroscience, Ramaiah Medical College and Hospitals, Bengaluru, India

**Keywords:** EEG, Entropy, Epileptic seizures, MLPNN classifier, Power spectral density, Teager energy

## Abstract

Detection of epileptic seizure activities from long-term multi-channel electroencephalogram (EEG) signals plays a significant role in the timely treatment of the patients with epilepsy. Visual identification of epileptic seizure in long-term EEG is cumbersome and tedious for neurologists, which might also lead to human error. Therefore, an automated tool for accurate detection of seizures in a long-term multi-channel EEG is essential for the clinical diagnosis. This study proposes an algorithm using multi-features and multilayer perceptron neural network (MLPNN) classifier. After appropriate approval from the ethical committee, recordings of EEG data were collected from the Institute of Neurosciences, Ramaiah Memorial College and Hospital, Bengaluru. Initially, preprocessing was performed to remove the power-line noise and motion artifacts. Four features, namely power spectral density (Yule–Walker), entropy (Shannon and Renyi), and Teager energy, were extracted. The Wilcoxon rank-sum test and descriptive analysis ensure the suitability of the proposed features for pattern classification. Single and multi-features were fed to the MLPNN classifier to evaluate the performance of the study. The simulation results showed sensitivity, specificity, and false detection rate of 97.1%, 97.8%, and 1 h^−1^, respectively, using multi-features. Further, the results indicate the proposed study is suitable for real-time seizure recognition from multi-channel EEG recording. The graphical user interface was developed in MATLAB to provide an automated biomarker for normal and epileptic EEG signals.

## Introduction

EEG is a clinical procedure carried out for monitoring, diagnosing, and determining neurological disorders related to epilepsy [1]. Epilepsy is a neurological disorder caused due to abnormal electrical discharges in the brain that are characterized by seizures and sudden changes in the electrical activity of the brain. An epileptic seizure is commonly identified as a slow-spike waveform. The unpredicted nature of these seizures makes the daily life immobile with temporary impairments of perception, speech, memory, consciousness and may lead to an increased risk of injury or death [2, 3]. Nearly 4% of world population experience seizure at some stage of their life out of which 1% are epileptic. In interictal recordings, epileptic seizures are usually activated with photostimulation, hyperventilation, and other methods. However, the drawback is that the behavior of provoked epileptic seizures is not necessarily the same as natural ones [4].

The long-term video-EEG recording is a significant milestone to not only capture and analyze ictal events but also help in the contribution of valuable clinical information. Traditional methods of analyzing EEG are time-consuming and a tedious job done by neurologists. Visual interpretation of these long-term EEG recordings can lead to human error and is inefficient [5]. Moreover, the EEG recordings of epileptic seizure are similar to the waves that are a part of background noise and artifacts. For these reasons, automated detection of epileptic seizures is needed to reduce the analyzing time and help the neurologists.

The brain is a nonlinear and complex dynamic system, so detecting seizures by a single-channel EEG is not sufficient. Thus, the processing of multi-channel EEG plays a vital role in seizure detection across the brain. However, multi-channel EEG signals impose the challenge of efficiently extracting useful information, and hence, only a few studies have focused on them [6, 7]. An ample number of studies have been proposed for seizure detection. Such technique involves preprocessing, feature extraction, and classification. Selecting significant features is essential to distinguish between normal and epileptic EEG signals. Our focus is on making the job of the neurological experts easy by making the abnormality visually understandable by using the multi-features extraction methods.

Multi-channel EEG recording plays a crucial role in recognizing the epileptic seizure activities from the brain lobes. Automated computed aided screening tool to help neurologist in saving their investigation period and enhance the required clinical diagnosis. Therefore, this study proposes the automated detection of epileptic seizures from multi-channel EEG recordings using multi-features. It also helps neurological experts have a complete picture of the epileptic EEG recordings preventing them from false alarms and leading to decision support with increased accuracy.

**Fig. 1:**
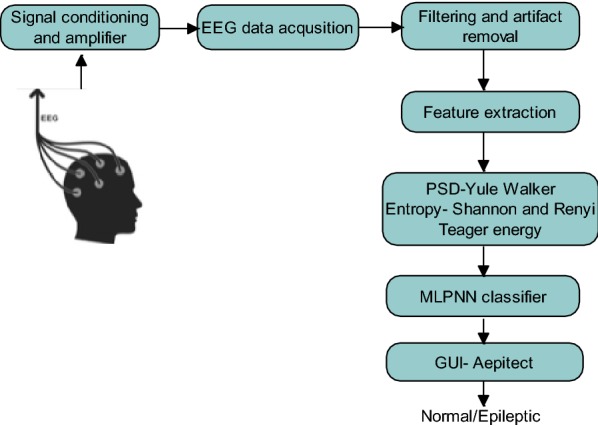
Block diagram of the proposed Aepitect technique

Figure 1 shows the flow of the proposed automated seizure detection system. The database was obtained after taking consent from the ethical committee. The raw data that were obtained had other noises such as power-line noise and motion artifacts other than EEG recording. Suitable filtering techniques were implemented to obtain clean EEG. The 50-Hz power-line noise was removed by using a notch filter, a bandpass filter had been implemented to get the signals in the range of 0.5–40 Hz, and independent component analysis (ICA) was applied to remove the motion artifacts.

The EEG data consisting of both normal and epileptic data annotated by the clinician were segmented separately for offline analysis. The features of interest to evaluate the epileptic EEG, namely PSD, entropy, and TE, were extracted, and descriptive analysis was carried out. The extracted features were given as input to the MLPNN binary classifier. Finally, a graphical user interface (GUI) has been developed to label the signals as normal or epileptic.

So far, several automated epileptic seizure detection methods have been proposed. In the early 1980s, the automated seizure detection procedure for a long duration of EEG recordings was initiated [8]. Guo et al. [9] proposed a line length of EEG as a feature and artificial neural networks classifier-based automated detection of epileptic seizure. The database considered was subjected to preprocessing, visual inspection, and artifact removal. EEG was decomposed into different sub-bands using discrete wavelet transform (DWT), and line length feature was extracted. The classification was done using a three-layer MLPNN, and a classification rate of more than 95% was achieved. Back-propagation neural network classifier with periodogram and autoregressive features was proposed [10]. Orhan et al. [11] used DWT-based features with MLPNN model for automated detection of epileptic seizures. Kamath [2, 3] proposed Teager energy as a quantitative feature for EEG signals. The study used the University of Bonn database to extract Teager energy and compared the classification outcome with Higuchi’s fractal dimension and sample entropy. It has been proved that TE provided an accuracy rate of 97.8%, and it can be used in real-time automated applications.

Gurwinder et al. [12] proposed a study to detect epileptic seizures using wavelet transformation and spike-based features. The work used University of Bonn database and wavelet transformation as its preprocessing technique. Spike-based parameters were extracted from both normal and interictal data. MLPNN was used for classification which gave an accuracy of 98.6%. Epileptic seizure detection method was developed using autoregressive modeling [13] and that showed the classification accuracy of 84.2% using MLPNN. Hierarchical EEG classification system using best basis-based wavelet packet entropy method was proposed [14]. Abbasi and Esmaeilpour [4] proposed a study to choose statistical characteristics of brain signals for detection of epileptic seizures using DWT and perceptron neural network. Their study used University of Bonn database and DWT as a feature extraction method. Statistical characteristics are derived, and a multi-perceptron neural network was used as a classifier which gave an accuracy of 98.33%.

The features such as mean, standard deviation, skewness, kurtosis, and the median in the first and second derivative of EEG signals were extracted for mobile-based automated epileptic seizure detection using k-means clustering technique [15]. Bogaarts et al. [16] extracted features such as curve length, root mean square, band power, zero crossing, Hjorth parameters, and Teager energy to classify epileptic EEG from normal using the support vector machine (SVM) classifier. Empirical mode decomposition (EMD) followed by DWT was applied on EEG signals to compute log energy entropy. The obtained features were classified using K-NN classifier, which yields the accuracy of 89.4% [17]. In the recent study [18], significant features were selected from neighborhood component analysis for the classification of focal and non-focal EEG signals. The highest classification accuracy of 96.1% was obtained using SVM classifier.

Other studies have introduced approximation entropy [19], log and norm entropy [20, 21], variants of entropies [22, 23], DWT-based features [24], time–frequency-domain features [25], Shannon, Renyi, log entropy and spectral entropy [20, 21], spectral and statistical features [26], and the classification of epileptic seizures. An optimal configuration of MLPNN was derived for the classification of epileptic seizures [27].

It was inferred from various studies that automated seizure detection was based on using single feature extraction. However, using multi-features would help in better classification of normal and epileptic data and classification accuracy.

## Materials and methods

### EEG data acquisition

The EEG recordings used in this study were obtained from Ramaiah Memorial College and Hospital, Bengaluru, after getting consent from the ethical committee. Unipolar multi-channel (19 channels) EEG recordings from 20 patients (11 male and nine female), each of 20-min duration, were considered for the study. International 10–20 system was used for the electrode placement, and data were recorded at a sampling rate of 128 Hz. The data, consisting of both normal and epileptic seizures annotated by clinician, were segmented separately for offline analysis. The 19 channels include the recordings from the following placement of the electrodes: Fp1, Fp2, F7, F3, Fz, F4, F8, T3, C3, Cz, C4, T4, P3, Pz, T6, O1, and O2. Table 1 shows each patient information used in our study.

**Table 1:** Information of each patient EEG data used in our work

Patient no.	Sex	Age	Seizure type
1	M	80	SPS, CPS, GTCS
2	F	20	SPS, CPS, MCS
3	F	5	SPS, CPS
4	M	11	SPS, CPS
5	F	12	CPS, GTCS
6	F	26	SPS, CPS, MCS
7	F	7	SPS, CPS, GTCS
8	M	3	SPS, CPS
9	M	12	SPS, CPS, MCS
10	M	44	SPS, CPS, GTCS
11	M	13	SPS, CPS, MCS
12	M	16	CPS, MCS
13	M	5	CPS, MCS, GTCS
14	F	7	SPS, CPS, GTCS
15	F	9	CPS, GTCS
16	M	6	SPS, CPS
17	M	5	SPS, CPS, GTCS
18	F	13	SPS, CPS
19	F	21	SPS, CPS, MCS
20	M	18	CPS, MCS

*M* Male, *F* Female, *SPS* Simple partial seizure, *CPS* Complex partial seizure, *GTCS* Generalized tonic–clonic seizure, *MCS* Myoclonic seizure

### Preprocessing

Suitable filtering techniques were introduced to eliminate noise and artifacts. An infinite impulse response (IIR) notch filter of order 2 was implemented to remove the 50-Hz power-line noise. A bandpass filter of order 5 with a higher cutoff frequency of 40 Hz and a lower cutoff frequency of 0.5 Hz was implemented to retain the EEG rhythms of interest in the data. The filter design specifications are: the passband ripple and attenuation in the stop band were set to 3 dB and 40 dB, respectively. Artifacts were removed from the filtered EEG using joint approximation diagonalization of eigen-matrices-based ICA technique [28–30].

### Feature extraction

Selecting significant features is essential for the proper classification of epileptic seizures. The number of extracted features should be less and easy to compute with reduced computational time. The significant characteristic of an epileptic EEG is a slow wave followed by a spike. The epileptic EEG varies significantly from that of a normal EEG in frequency, period, complexity, etc. Considering all these parameters, the following features were selected for our research work: power spectral density, entropy (Shannon and Renyi entropy), and Teager energy [19–23].

In this paper, PSD was used as the power of the EEG signal increased during epileptic activity. Entropy is a measure of the complexity or uncertainty of a signal, higher during epileptic activity, and gives a clear distinction between normal and epileptic. Teager energy depends on the amplitude of the epileptic data that is higher than that of the normal signal. From the preliminary study, it was identified that PSD using Yule–Walker method showed better results as compared to the other methods of PSD like Welch method, Burg’s method, and Thomson’s method.

#### Yule–Walker method

Yule–Walker method is an autoregressive (AR) method that estimates spectra with narrow peaks by placing the poles of the polynomial close to unity. Narrowly banded spectra are quite common in practice. Hence, this has been chosen as the best method for feature extraction for the study. The AR parameters are represented as *θ* by forming a biased estimate of the signal’s autocorrelation function and a minimization of a prediction error [31].

For this study, a fourth-order autoregressive model was used to produce the PSD estimates. The preprocessed signal was segmented at a length of 0.5 s, followed by obtaining the PSD estimates, and then, the maximum PSD of each segment was determined. This process was carried out in the complete study.

#### Shannon entropy

It is a measure of the randomness or disorder in physical systems or the amount of average information gained by observations of disordered systems. It is the best possible lossless compression and gives low entropy values for varied distribution and high entropy values when outcomes are uniformly distributed. Shannon’s entropy is given by the equation [32]:

1 $$E = - \sum p_{i} \log_{2} p_{i}$$

where $pi$ is the probability of occurrence of the signal.

#### Renyi entropy

It is a generalized form of Shannon’s entropy when the Renyi estimation factor $\alpha = 1$. It is also called quadratic entropy as $\alpha = 2$. The value of α is estimated to be taken as 2 as peak accuracy is achieved with specificity higher than for Shannon’s case. Renyi’s entropy equation is given as [33]:

2 $$E = \frac{1}{1 - a}\sum \log_{2} p_{i}^{a}$$

where $$\alpha \ge 0$$ and $$\alpha \ne 1$$.

There are a few special cases in case of Renyi entropy. They include:

- $\alpha = 0$—obtains maximum entropy.
- $\alpha = 1$—recovers the Shannon’s entropy.
- $\alpha = \infty$—obtains the minimum entropy.

It can also be added that when α has larger positive value, it is sensitive to events that occur often and when α has larger negative value, and it is sensitive to events that occur seldom.

Since entropy is a function of probability, in this study, the probability was estimated using the histogram method by setting the bins with a uniform width.

#### Teager energy

Teager energy is a nonlinear operator, which can be used for energy estimation of a non-stationary signal. This feature is extremely sensitive to amplitude and frequency changes of a signal. The method is computationally very efficient, as it requires only three samples at any given instance to calculate the physical energy. Since the EEG signal is non-stationary, Teager energy operator can be used as a discriminating feature for normal and epileptic data set.

As per the Teager algorithm, the Teager energy (TE) is estimated from the signal *x(n)* through the formation of time-delayed state-spaced vectors *x*(*n*) = [*x*_1_, *x*_2_, *x*_3_,…, *x*_*n−1*_, *x*_*n*_] where n is the data points as follows [34]:

3 $${\text{TE}} = \frac{1}{N - 1}\mathop \sum \limits_{n = 2}^{N - 1} x_{n}^{2} - x_{n - 1} *x_{n + 1}$$

where *N* is taken to be 64 (segmentation length of 0.5 s).

From the equation, it is clear that Teager energy takes into account the amplitude and the corresponding frequency to determine the physical energy.

### Descriptive analysis

Descriptive analysis was performed on the extracted feature samples obtained from epileptic and normal data. The mean, standard deviation (SD), minimum, maximum, interquartile range (IQR), first quartile (Q1), median (Q2), third quartile (Q3), and semi-interquartile deviation (SID) were estimated for extracted features using box plot. The *p* and *z* values were found for individual patients for normal and epileptic feature values. The *p* value should be less than 0.05 which gives a confidence level of greater than 95%, and the *z* value should be less than 1.96 and greater than − 1.96 [34]. The descriptive analysis of extracted features showed that the obtained features are significant for further analysis.

### Classifier

MLPNN is a feed-forward neural network, which was used for binary classification of the EEG signal. It contains three consecutive layers, namely input, hidden, and output layer [35–38]. In this study, we have used the MLPNN model with a single hidden layer of 10 neurons. Hyperbolic tangent and tangent sigmoid were used as input to hidden and hidden to output activation function, respectively. A scaled conjugate gradient back-propagation was used as a training function. The classification target was set to 0 for normal and 1 for epileptic [27].

### Performance evaluation

The performance of the proposed method was evaluated based on the sensitivity (*S*^+^), specificity (*S*^−^), and false detection rate (FDR) for individual patients as follows [11, 19, 25]:

4 $$S^{ + } = \frac{{{\text{Correctly}}\,\,{\text{detected}}\,{\text{epileptic}}\,{\text{seizures}}}}{{{\text{Total}}\,{\text{number}}\,{\text{of}}\,{\text{epileptic}}\,{\text{seizures}}}}$$

5 $$S^{ - } = \frac{{{\text{Correctly}}\,\,{\text{detected}}\,{\text{normal}}\,{\text{activities}}}}{{{\text{Total}}\,{\text{number}}\,{\text{of}}\,{\text{activities}}}}$$

6 $${\text{FDR}}({\text{per}}\,{\text{hour}}) = \frac{{{\text{Number}}\,{\text{of}}\,{\text{false}}\,{\text{detections}}}}{{{\text{Total}}\,{\text{length}}\,{\text{of}}\,{\text{the}}\,{\text{data}}}}$$

## Results

**Fig. 2:**
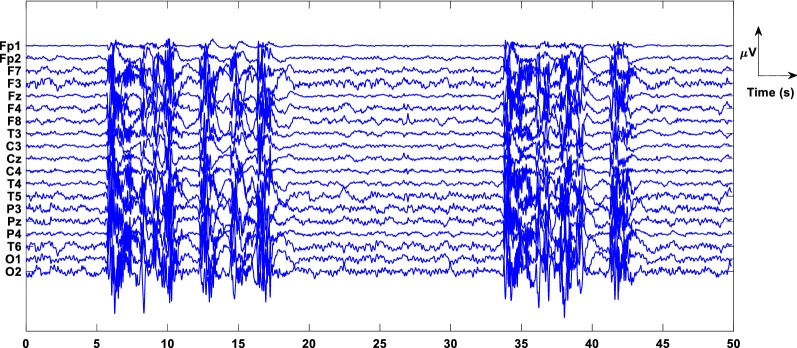
Preprocessed multi-channel EEG signal

This study takes into account 20 patients’ multi-channel EEG recordings. Notch filter and bandpass filters with appropriate cutoff frequencies were used to remove line noise of 50 Hz and other background noises. ICA was used to remove motion artifacts, and artifact-removed multi-channel EEG is shown in Fig. 2. To maintain the uniformity of the signal, the EEG was segmented at 0.5 s duration.

A descriptive analysis of the obtained feature from epileptic and normal data was performed. Table 2 shows that the statistical parameters for both epileptic and normal EEG samples obtained from patients were highly distinguishable. Results show that PSD, entropy, and Teager energy in epileptic EEG were more compared to that of normal EEG. A *p* value was found between normal and epileptic extracted feature samples using a two-sided Wilcoxon rank-sum test. For all the features, *p* value was found to be less than 0.05 and *z* value greater or lesser than the prescribed limits, which indicates that all the features were suitable for classification. Table 2 shows the obtained *p* and *z* values (Table 3).

**Table 2:** Descriptive analysis of extracted features

Feature	EEG	Mean	STD	Min	Q1	Q2	Q3	IQR	Max	SID
Yule–Walker PSD	N	0.6027	1.0179	0.0018	0.1536	0.3402	0.5719	0.4183	15.063	0.2091
	E	20.866	19.7967	0.1609	7.0496	15.703	27.513	20.4636	259.5852	10.2318
Shannon entropy	N	3.2285	0.5698	1	2.9579	3.4082	3.6565	0.6986	4.1804	0.3493
	E	2.0793	0.5666	0.9016	1.6943	2.0441	2.4196	0.7252	3.8029	0.3626
Renyi entropy	N	3.4005	0.7148	0.8493	3.1329	3.4929	3.8295	0.6966	5.3897	0.3483
	E	5.8863	0.5731	2.9858	5.5831	6.1188	6.2974	0.7142	6.7119	0.3571
Teager energy	N	0.7101	2.9253	0.0012	0.0719	0.1917	0.4419	0.3700	80.621	0.1850
	E	19.24708	51.654	0.0337	3.6780	7.0098	13.378	9.7003	720.1391	4.8501

‘N’ stands for normal and ‘E’ stands for a person with epilepsy

**Table 3:** Wilcoxon rank-sum test results

Feature name	*p* value	*z*-score
Yule–Walker PSD	< 0.05	− 50.8174
Shannon entropy	< 0.05	49.87157
Renyi entropy	< 0.05	− 51.2641
Teager energy	< 0.05	− 51.2896

The classifier was trained using holdout cross-validation method with the ratio of 70 − 30 used for training and testing. Highest sensitivity and specificity of 86.2% and 95.2% were obtained using Renyi entropy for individual features, respectively. Further, sensitivity, specificity, and FDR of 97.8%, 96.4%, and 0.15 h^−1^ were recorded using multi-features which were highest than all other combinations. Table 4 shows the classification results of the proposed system for all individual features and multi-feature combination.

**Table 4:** Epileptic seizure detection results using the proposed method

Feature name	*S* ^+^	*S* ^−^	FDR (h^−1^)
Yule–Walker PSD	86.5	94.4	3
Shannon entropy	86.9	80.0	3
Renyi entropy	96.2	95.2	2
Teager energy	82.0	94.8	3
Multi-features	97.8	96.4	1

**Fig. 3:**
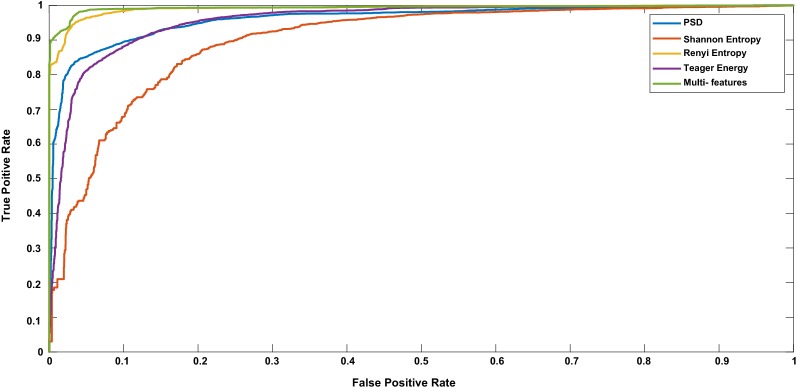
ROC curve for different features obtained from EEG data

Figure 3 shows the ROC curve obtained from the classification results of PSD, Shannon entropy, Renyi entropy, Teager energy, and multi-features. Maximum AUC of 0.97 was obtained for multi-features, whereas a minimum of 0.83 attained for Shannon entropy. Classification results revealed that the highest performance measures were achieved using multi-features than the single features with the betterment of sensitivity, specificity, and FDR.

## Discussion

**Fig. 4:**
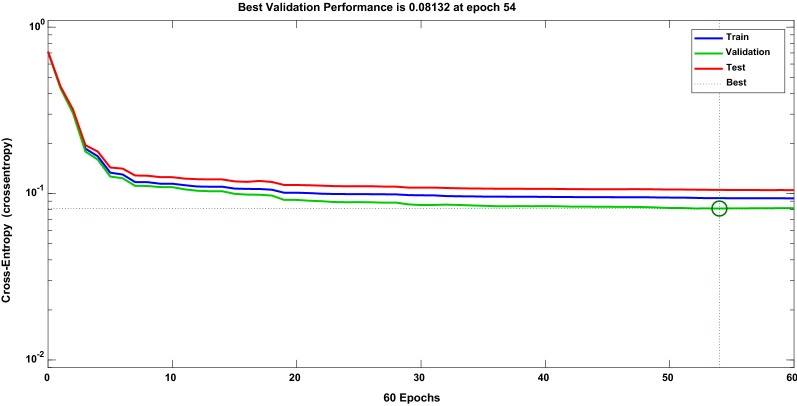
Showing best validation performance at epoch 60

**Fig. 5:**
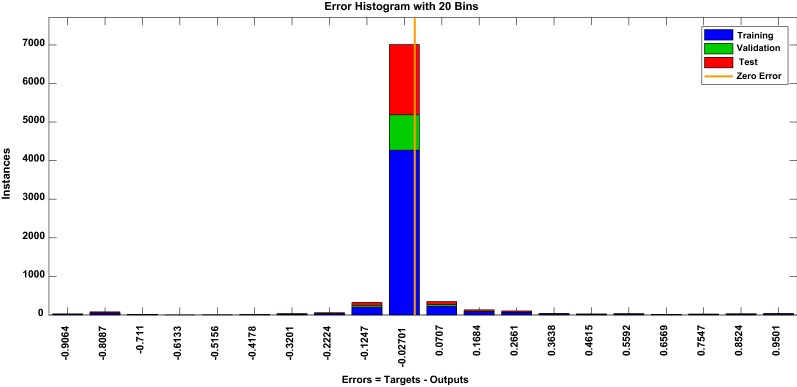
Histogram of each testing validation and training state

The foremost objective of this study was to introduce an automated detection of epileptic seizures using multi-channel EEG. Four features, namely PSD, variants of entropy, and Teager energy, were utilized followed by MLPNN classifier. These features were selected for the study based on previous performance on other databases. Experimental results show that multi-features perform better as compared to single features. Figure 4 shows the best validation performance of MLPNN classifiers for multi-features. It can be seen that the best validation performance of 0.08 was obtained at epoch 54. Further, Fig. 5 shows the error histogram of training, validation, and the testing state. As it can be seen, error difference between target and predicted values is minimum, exhibiting the good convergence.

**Fig. 6:**
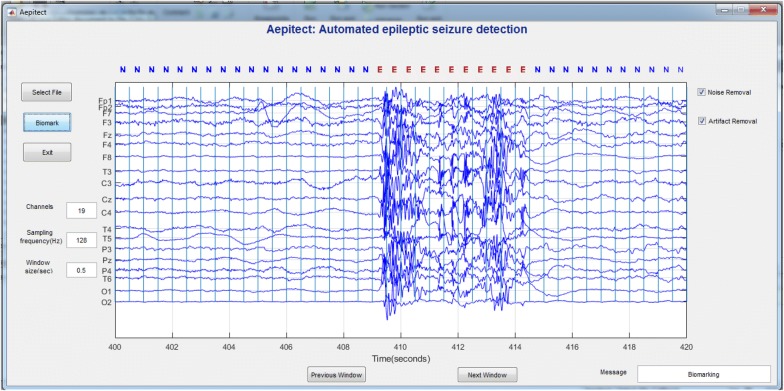
Screenshot of GUI referred as ‘Aepitect’ developed in MATLAB

A GUI was built using MATLAB for automated classification of epileptic seizures using the trained model developed. The name assigned to the GUI developed was ‘Aepitect’, which stands for automated epileptic seizure detection. The GUI was designed in such a way that it displays the 20 s of EEG every page. Features were extracted at a segmentation length of 0.5 s, and the same were used to classify using the trained model. Figure 6 shows the screenshot of ‘Aepitect’, and it was cross-validated with the neurologist and found 98.5% matching.

The button ‘Select File’ allows the user to select the patient file, and the button ‘Biomark’ performs preprocessing, feature extraction, classification, and biomarking.

The performance of the proposed approach was compared with the other existing studies reported earlier. Table 5 shows the comparison results between different studies. As it is seen from Table 5, most of the studies have used single-channel EEG data from the University of Bonn and achieved better results. One should take the attention while comparing the performance of different methods since different EEG databases were used in their respective studies. University of Bonn database was found to be clean EEG, and it works well for all the methods. However, the challenge arises while dealing with long-term multi-channel EEG. Therefore, we have used our database for the study to overcome the existing issues such as less sensitivity, specificity, and FDR.

**Table 5:** Comparison results of some epileptic seizure detection methods

Author	Features	Classifier	Results	Database
Kiymik et al.	Autoregressive features	Back-propagation neural network	Accuracy 95%	Neurology department of the Medical Faculty Hospital of Dicle University
Orhan et al.	DWT-based features	MLPNN	Accuracy 99.6	University of Bonn
Kamath 2013	Teager energy	Radial basis function neural network	Accuracy 97.8%	University of Bonn
Gurwinder et al. 2015	Wavelet transformation and spike-based features	MLPNN	Accuracy 98.6	University of Bonn
Ahammad et al.	Energy, entropy, standard deviation, maximum, minimum, and mean	MLPNN	Accuracy 84.2	University of Bonn
Wang et al. 2011	Wavelet packet entropy	K-NN	Accuracy 100%	University of Bonn
Abbasi et al. 2017	DWT-based features	MLPNN	98.33%	University of Bonn
Srinivasan et al. 2007	ApEn	Recurrent Elman neural network	Accuracy 100%	University of Bonn
Proposed method	PSD, entropy, and Teager energy	MLPNN	Sensitivity 97.8% Specificity 96.4% FDR 1 h^−1^	Ramaiah Memorial College and Hospital, Bengaluru

The results of seizure detection algorithms are usually evaluated based on the sensitivity of the raised alarms (number of detected seizures/total number of seizures) and false detection rate; it is not evaluated by the sensitivity and specificity of epochs/segments. It was noticed that studies using University of Bonn database had classified epileptic seizures as epochs/segments instead of detecting them as a complete seizure. When comparing with other methods, our method follows the evaluation criteria of sensitivity and FDR to evaluate the performance of the algorithm. As compared to other methods listed in Table 5, the proposed method matches the results of other studies without using any DWT on EEG signal.

The significant contributions of the proposed study were:

1. EEG data were recorded at Ramaiah memorial hospital, Bengaluru, and were used for the study.
2. Artifacts were removed automatically using ICA technique and experts validated same at Ramaiah memorial hospital, Bengaluru.
3. From the preliminary study, the best PSD method (Yule–Walker) was selected for the feature extraction.
4. Three features, namely PSD, variants of entropy, and Teager energy, were used for the feature extraction.
5. The descriptive analysis shows the noticeable band difference between normal and epileptic EEG activities.
6. Wilcoxon rank-sum test shows the evidence to reject the null hypothesis at the 5% significance level.
7. Classification results show the better performance using multi-features as compared to the single features.
8. A MATLAB GUI called ‘Aepitect’ was developed for automated detection.

The above findings suggest that the proposed method is suitable for automated detection of epileptic seizures in real time. The complete study was implemented in MATLAB 2016b using 8 GB RAM, CPU 2 GHz with Intel i5 processor. As a future step, more features will be included to increase the sensitivity and decrease the FDR. Further, deep learning concept will be explored for the classification of epileptic seizures.

## Conclusion

This study provides a multi-channel EEG analysis for the detection of epileptic seizures using PSD, entropy, Teager energy, and MLPNN classifier. Initially, EEG signals were preprocessed to remove noise and artifacts, and features were extracted. Descriptive analysis and Wilcoxon rank-sum test proved the suitability of the extracted features for classification with noticeable band difference between normal and epileptic EEG. The simulation results showed sensitivity, specificity, and false detection rate of 97.8%, 96.4%, and 1 h^−1^, respectively, using multi-features. Results indicate that the proposed study is suitable for real-time seizure recognition from multi-channel EEG recording. The graphical user interface referred as ‘Aepitect’ was developed in MATLAB to provide an automated biomarker for normal and epileptic EEG signals. It is anticipated that the proposed algorithm will offer a faster and accurate diagnosis and also reduce the time spent on detecting seizures from long-term multi-channel EEG recordings and can be extended to more patients for long-term EEG.
